# Applying User-Centered Design Methods to Understand Users' Day-to-Day Experiences Can Inform a Mobile Intervention for Binge Eating and Weight Management

**DOI:** 10.3389/fdgth.2021.651749

**Published:** 2021-06-04

**Authors:** Andrea K. Graham, Sarah W. Neubert, Angela Chang, Jianyi Liu, Emily Fu, Emilie A. Green, Rachel Kornfield, Jennifer Nicholas

**Affiliations:** ^1^Center for Behavioral Intervention Technologies, Northwestern University Feinberg School of Medicine, Chicago, IL, United States; ^2^Department of Medical Social Sciences, Northwestern University Feinberg School of Medicine, Chicago, IL, United States; ^3^Weinberg College of Arts and Sciences, Northwestern University, Evanston, IL, United States; ^4^Department of Psychiatry and Behavioral Science, Northwestern University Feinberg School of Medicine, Chicago, IL, United States; ^5^Department of Preventive Medicine, Northwestern University Feinberg School of Medicine, Chicago, IL, United States; ^6^Orygen, Melbourne, VIC, Australia; ^7^Centre for Youth Mental Health, The University of Melbourne, Melbourne, VIC, Australia

**Keywords:** user-centered design, obesity, binge eating, mobile intervention, personas

## Abstract

**Introduction:** Weight loss apps to date have not directly addressed binge eating. To inform the design of a new mobile behavioral intervention that addresses binge eating and weight management, we applied user-centered design methods to qualitatively assess how target intervention consumers experience these conditions in their day-to-day lives.

**Methods:** The participants were 22 adults with self-reported obesity (body mass index ≥30) and recurrent binge eating (≥12 episodes in 3 months) who were interested in losing weight and reducing binge eating. The participants completed a digital diary study, which is a user-centered design technique for capturing individuals' day-to-day experiences in relevant contexts. Qualitative data describing the participants' experiences with binge eating and obesity were analyzed using thematic analysis. The results were then used to create personas (i.e., character archetypes of different intervention consumers).

**Results:** The participants described triggers for binge eating and indicated that binge eating and excess weight negatively impact their mental health, physical health, and quality of life. The resulting personas reflected five different struggles individuals with these health problems experience in managing their binge eating and weight.

**Conclusions:** Individuals with binge eating and obesity have varying precipitants of problematic eating as well as varying motivations for and challenges to behavior change. To meet the needs of all who seek intervention, an ideal intervention design will account for variations in these factors and be relevant to diverse experiences. Insights from the diary study and resulting personas will inform the next phases of the user-centered design process of iteratively designing prototypes and testing the intervention in practice.

## Introduction

Up to 30% of treatment-seeking individuals with obesity engage in binge eating (1–3), and the majority (>75%) of people with recurrent binge eating also have overweight or obesity (4). We are designing a new mobile behavioral intervention for people with obesity and recurrent binge eating who want to lose weight and reduce their binge eating, and intervention targets will be drawn from evidence-based psychological and behavioral principles for managing weight and binge eating. An intervention for obesity and binge eating is needed because the psychological or behavioral interventions that have been tested to date have not produced substantive changes in both of these outcomes (5, 6). Moreover, to our knowledge, weight loss apps have not directly addressed binge eating. This is problematic because, without adequate attention to the mechanisms that drive and maintain binge eating, some behavioral recommendations for weight loss (e.g., dietary restriction) can exacerbate eating disorder behaviors. Therefore, specialized intervention that concurrently addresses obesity and binge eating is warranted.

Digital interventions offer an exciting opportunity to increase access to care and extend the reach of services to individuals when they are needed most (7). We are using a mobile format so that our new intervention can scale to the substantial number of individuals in need of services (2, 4). However, to ensure that our intervention will be used in the moments and contexts when it is needed most, it must be designed to reflect the needs of individuals with obesity and binge eating and address the diversity of experiences these individuals face in trying to manage their eating and weight. Too often, digital health interventions have been designed based on what clinicians think intervention consumers need without sufficiently accounting for what they want (8, 9). This approach has been problematic as consumers do not use the interventions (10), and consistent examples also indicate challenges with their successful implementation (9). To rectify these problems, clinical scientists need innovative methodologies for designing digital interventions that achieve high engagement and clinical significance.

We are taking a user-centered design approach to create this new intervention. User-centered design is a methodology that focuses on engaging deeply with end-users (i.e., target users of an intervention) to create technological solutions that are grounded in information about the individuals who will be using them and the settings in which they will be deployed (11–13). By putting end-users at the center of the design process, this methodology results in technologies that are easier to understand and use (11–13). From a clinical standpoint, our new mobile intervention is being designed to address five putative intervention targets that are theorized as mechanisms that contribute to a cycle of binge eating and changes in weight: overvaluation of weight and shape, unhealthy weight control practices, negative affect, dietary intake, and physical activity. To address these targets, the intervention will draw on evidence-based principles from behavioral weight loss treatment and cognitive behavioral therapy for binge eating (14–16). However, *how* the intervention is designed to address these intervention targets, so that it is effectively delivered in a mobile format, requires collaboratively involving end-users in the design process.

The first step in the user-centered design process is to conduct a needs assessment. A needs assessment is an essential aspect of design. By investigating the needs, goals, and preferences of end-users, we can ensure that a technological solution is designed to address a problem or fill an unmet need. Despite a robust literature documenting problems associated with binge eating and weight [e.g., (6, 17)] and the types of behavioral and cognitive strategies that would likely lead to improvement (14–16), we need a better understanding of how individuals with obesity and binge eating experience these problems in their day-to-day lives. Deeply understanding individuals' experiences—and the motivations and barriers that they face in managing these health problems—can inform how a mobile intervention could be designed to fit into these individuals' daily routines.

A variety of methods can be used to conduct a needs assessment, such as interviews, focus groups, and observations (12). To design our intervention, we used a method called a digital diary study, the purpose of which is to learn about and understand people's experiences as they occur in the context of their day-to-day lives. A diary study entails prompting users at various moments to share details about a particular experience. The digital format means that the users submit their entries *via* an online/mobile platform (as will be described in the “METHODS”). Various response formats can be administered in a diary study to collect qualitative and/or quantitative data (e.g., video recordings, uploading photographs, open-ended text responses, multiple choice, and yes/no questions). This study analyzed qualitative data from video recordings and open-ended text responses.

Data from a needs assessment are then used to inform the next step in the user-centered design process: ideation. Ideation is the brainstorming phase, when ideas and concepts for the intervention are generated. Brainstormed ideas are then used to create initial intervention designs. One method used in the ideation phase that can help inform intervention ideas is creating personas. Personas are composite archetypes of different potential intervention users, presented as distinct individual users, that show the diversity of perspectives that need to be considered in an intervention design (18, 19). Personas represent broad segments of actual users through detailed descriptions of hypothetical users. Created based on aggregated data from multiple potential users (20, 21), such as captured through a needs assessment, personas are presented as individual people and convey specific details about them, such as personal characteristics, motivations and goals, needs, and expectations. Personas help designers move from understanding users' needs to brainstorming and designing solutions that meet these needs. The value of personas is in serving as a reference point that designers can prospectively use to inform, justify, and build consensus around design decisions as well as assess that iterative designs reflect their intended users (18). Although creating personas cannot ensure that the eventual intervention yields high engagement, the use of personas helps ensure that interventions respond to the diversity of experiences and needs within intended user populations (22), which, in turn, may lead to heightened engagement.

Given these advantages, persona use is growing within behavioral health [e.g., (23–25)]. For example, personas have been developed to reflect hypothetical users of a weight loss coaching app (26). In that study, five personas were created, which reflected nuanced descriptions of different end-users' motivations to change their behaviors, the struggles they face in the process of losing weight, and the perceptions they have about their behavior and weight. Each persona described the hypothetical user's approach and readiness to engage in behavior change around losing weight, with a focus on their level of autonomy to pursue behavioral changes. The authors concluded that creating the personas enabled their designers to empathize with the struggles that individuals face in changing their behavior and to understand the variety of factors that must be addressed in a coaching app to help move people toward changing their behavior.

Despite the benefits of personas to the process of designing and validating a technology, to our knowledge, personas have not been applied to individuals struggling with binge eating and seeking weight loss nor to binge eating in general. Thus, the purpose of this paper is to (a) disseminate results from a digital diary study needs assessment with end-users that aimed to answer the research question of how individuals with obesity and binge eating experience these problems in their day-to-day lives and (b) present five personas that were created based on the insights that emerged from data analysis. Design methods are being increasingly used for health-related interventions [e.g., (26–31)], though they have been largely underutilized in the clinical science field (32) and, to our knowledge, rarely represented in eating disorders [cf (33, 34)]. Therefore, this paper offers an important example of the application of user-centered design methods to mobile behavioral intervention design. This work has relevance for informing future approaches to designing digital mental and behavioral health interventions.

## Methods

### Participants

The participants were non-pregnant, English-speaking adults with obesity [body mass index (BMI) ≥30 based on self-reported height and weight] and self-reported recurrent binge eating (≥12 binge eating episodes in 3 months). Binge eating was defined as “when someone eats an unusually large amount of food and feels a sense of loss of control while eating” (35). The eligible individuals were also interested in losing weight and reducing their binge eating. Among those eligible to participate, the final sample was selected to ensure a diverse cohort of target intervention users based on race/ethnicity, sex, and age. Specifically, the participants were purposely selected across a range of ages and, for race/ethnicity, in proportions approximate to the racial/ethnic distribution of individuals in Chicago according to 2010 census data (36) where future end-users were planned to be drawn for efficacy testing. More females than males were selected because the prevalence of binge eating is higher in females than in males (37).

Twenty-five individuals were invited to participate, of whom 22 completed the study and were included in these analyses. Though small for clinical research, this sample size was anticipated to be sufficient to achieve saturation (38) and is consistent with research in the field of human–computer interaction (39).

### Procedure

The study procedures occurred entirely online using dscout, a digital platform for qualitative and market research that enables capturing of the participants' in-the-moment, in-context experiences over time (40). Interested individuals responded to a study advertisement and completed a screener in dscout to assess eligibility. The invited participants then completed a digital diary study over 1 month that focused on understanding how binge eating and weight impact the participants' day-to-day lives, factors that trigger binge eating, who and what motivates them to work on their binge eating and weight, and the strategies that they use in their daily lives to manage their binge eating and weight.

The study was administered over nine parts. The participants submitted entries in response to each part, and the number of entries requested for each part varied depending on the research questions entailed (range = 1–4 entries per part; total entries = 14). For each part, the participants were asked to submit their entry or entries at any time of their choosing within the requested time window for that part (range = 2–7 days). Entries could be submitted from the participants' smartphones, and each entry took only a few minutes to complete. The participants were sent reminders through the platform to complete the entries before the scheduled end-date for each part. Parts were sequentially made available so that the participants focused on only one at a time.

Seven of the nine parts contained video entries (one per part) and open-ended text responses (range = 1–4 per part). For example, in the first part, the participants were asked to record a video while answering the questions “How do binge eating and weight impact your day-to-day life? Do you see these two things as related to each other, and if so, how?” The complete assessment guide is available upon reasonable request from the corresponding author.

The participants who completed all study procedures were compensated $100; all 22 participants included in this analysis were compensated. This study was approved by the Northwestern University Institutional Review Board, and all participants provided online informed consent.

### Analyses

To answer the research question of how individuals with obesity and binge eating experience these problems in their day-to-day lives, the participants' video recordings and open-ended text responses were analyzed. Video recordings were automatically transcribed by the dscout platform and edited for accuracy and deidentification by a study team member. Thematic analysis was used to analyze the data, following Braun and Clark's methodology (41). This involved becoming familiar with the data by reviewing the transcripts, using open coding to create codes, applying the codes to the transcripts, and finally organizing the codes into broader themes. Data were analyzed by two raters, one of whom was a licensed clinical psychologist with expertise in treating binge eating and obesity, and the other was an undergraduate student on the pre-medical track, majoring in biology and Spanish. After independently reviewing the data, the raters each identified a set of codes and then met together to discuss the codes and reach consensus on a codebook. The raters then reviewed the transcripts independently and met frequently to resolve questions about coding *via* consensus and to refine the codebook as needed.

After the data were analyzed, personas were created. This process began by identifying commonalities across participants' experiences that demonstrated representative struggles people had in managing their binge eating and weight. More specifically, by gathering a detailed understanding *via* the needs assessment of how individuals experience obesity and binge eating in their day-to-day lives, we learned overarching themes about factors that drive (precipitants) and result from (impacts) binge eating and obesity. Based on these themes, we identified patterns that represented different struggles that individuals experience in managing these problems—which are essential to delineate for understanding how to design a tool that could help someone change their behaviors. These patterns became our five personas. As details for the personas were created, the two raters revisited the transcripts to check and ensure that details about each persona were based on the participants' data from the diary study and that the five personas were comprehensive representations of the data.

Consistent with persona design (18, 22, 42), each of our personas was designed as a hypothetical user; no persona reflected a single participant in the digital diary study. Although a variety of features can be included in a persona, it was decided that these personas would include a name that encapsulates the key factor for why that hypothetical user struggles with managing their binge eating and weight, a brief description, a quote that demonstrates a statement this hypothetical user would make about their struggles, their motivations for and barriers to changing their behavior around eating and weight management, and an image that captured a precipitating factor associated with binge eating for that hypothetical user.

## Results

The 22 participants self-identified as primarily female (64%), with a mean age of 37.0 (SD = 10.2) years. The participants self-identified themselves as per the following race/ethnicities: 32% non-Hispanic White, 27% Black, 27% Hispanic, 9% Asian/Pacific Islander, and 5% unknown. At the start of the study, the participants reported an average BMI of 37.1 (SD = 5.4) and having engaged in an average of 20.5 (SD = 7.3) binge episodes over the previous 3 months. All the participants reported prior attempts to lose weight, and 91% reported prior attempts to stop binge eating.

### Precipitants of Binge Eating and Obesity

The participants described a multitude of factors that precipitated binge eating, which were grouped into two themes: internal (psychological and physical health) states and situational factors.

#### Internal States

The most common internal trigger for binge eating was emotions, such as anxiety, stress, and low mood. Boredom also created problems for the participants, as they stated that this led to binge eating or eating unhealthy foods:

“When I get bored, sometimes I crave snacks. Instead of having a healthy snack, I eat unhealthy.” [Participant (Pt) 19]

Physical health problems and sleep issues contributed to binge eating and unhealthy eating. One participant cited lack of sleep as problematic for making healthy decisions:

“Lack of sleep and anxiety are definite triggers. Not getting enough sleep makes making smart decisions so much harder.” (Pt 5)

Pre-occupation with food or hunger was also problematic. In these instances, the participants described feeling very hungry and feeling unable to think about things besides eating, which could result in binge episodes:

“I really want to eat something, and I'm really hungry. […] When you're really thinking about food, everything is food.” (Pt 14)

The participants described a perceived need to eat food that was available, regardless of their hunger or interest in the food. In these instances, they felt unable to avoid binge eating:

“Even though the foods were ‘healthy,’ I kept asking myself ‘why?’ I KNEW I wasn't hungry, but just the thought of a tuna sandwich and a bag of chips after the banana was too much to resist.” (Pt 22)

In fact, some participants anticipated and therefore planned for a binge episode:

“I hate to say it and admit it, but I'm getting ready to do some bingeing. Because I'm in whole foods, I'm getting ready to be at the dessert counter, and just, I'm just buying stuff.” (Pt 9)

Such experiences were also related to difficulties with setting or maintaining goals for healthy eating, which could result in problems with motivation that led to binge eating. For example, a participant described that when they failed to achieve certain health-related goals, they would abandon their plans and engage in unhealthy or binge eating:

“It (binge eating) started with creating an expectation for myself that I wasn't able to achieve. When I wasn't seeing the results I wanted, I would develop an ‘oh well, it doesn’t even matter' mentality.” (Pt 13)

The results in this theme together indicate that it will be important for an intervention to help individuals identify unhelpful patterns of thinking, address negative affect, and learn strategies to change their behaviors in response to these triggers.

#### Situational Factors

Triggers were also commonly driven by situational factors. Situational triggers included factors such as eating alone, engaging in eating-related activities (e.g., events involving food), having access to certain foods, and experiencing interpersonal problems. Late night binge eating was common for the participants. The participants also reported that problematic eating was exacerbated by an unstructured eating pattern. For example, this participant failed to eat much during the day, which led to binge eating that evening:

“Binge eating for me is triggered by a necessity to eat late at night and then trying to eat all of my meals at one time, feeling like I missed out on things because I go the whole day without eating.” (Pt 9)

Another example in which unstructured eating created problems was through too much snacking as described by this participant:

“I snack at the weirdest times and throughout the day, primarily eating the wrong snacks.” (Pt 3)

Eating outside the home, such as eating at fast-food establishments or from a vending machine, was a trigger for unhealthy or binge eating as described by this participant, for whom eating outside the home resulted in secretive eating, which is a hallmark feature of binge eating:

“I'm tired and I just want to stop at the nearest place and eat, and then eat dinner when I get home as well. Because I will make sure to not tell my husband that I stopped.” (Pt 8)

Conversely, some participants described difficulties in sustaining a healthy pattern of eating given a perception that their home environment hindered healthy behaviors. In this example, the participant's partner purchased unhealthy foods, making it harder for the participant to sustain healthy eating:

“I had a miscommunication with my wife. (…) When I was explaining to her we need to get fruit and vegetables, (…) she ended up getting me chicken and cookies instead. And so, I was really trying to get my family to support me with binge eating, but it didn't work.” (Pt 1)

An understanding of precipitants is crucial for informing points for intervention. The results here indicate the powerful role that both internal and external factors can play in binge eating. Thus, an ideal intervention will be designed to account for variation in these factors.

### Consequences of Binge Eating and Obesity

In understanding how the participants experience binge eating and obesity in their day-to-day lives, themes also emerged on the significant and impairing impact binge eating and obesity have on the participants' physical health, mental health, and quality of life.

#### Impact on Physical Health

The participants commented on the physical health impact of obesity and binge eating, such as feelings of pain or soreness in specific body parts like their knees, hips, and back. Though physical problems were often attributed to excess weight, some participants acknowledged the role of binge eating in causing health problems through weight gain. For example, this participant described how binge eating “too many snacks and junk food” had “impacted my midsection” and caused other health problems:

“I used to just be in such good shape to run around with my kids or play basketball and things. But now I just find myself low energy and oftentimes I have trouble sleeping. And so I just really think the binge eating is affecting my overall health.” (Pt 25)

The participants also described how their weight makes it harder for them to engage in physical activities, like walking, as noted by this participant:

“(When) you don't feel like you're at the proper weight it makes things difficult. It makes it harder for you to go up a flight of stairs, walk for long distances, stand for long periods of time.” (Pt 9)

The participants said that binge eating can result in feeling fatigued afterward. Additionally, the participants were impacted by knowing other family members who struggle with diabetes, indicating concerns or motivations to lose weight to reduce their own risk of diabetes based on their family history as shared by this participant:

“I am trying to lose weight mainly to reduce my chances of becoming a diabetic by a lot, because diabetes is genetic in my family and I have been a borderline diabetic multiple times.” (Pt 24)

#### Impact on Mental Health

The participants' responses reinforced the significant impact that obesity and binge eating have on their mental health. They described feelings of guilt, failure, and low mood that can accompany and prompt episodes of binge eating as well as the ways in which excess weight contributes to low self-esteem. One participant described how binge eating serves as a coping strategy during episodes of depression while recognizing that these eating episodes perpetuate a negative cycle:

“My depression came back, and I guess bingeing is the only thing that I guess helps me get through. But when I get through binge eating, then I'm just even more depressed.” (Pt 10)

Indeed this example shows how episodes of binge eating yield strong negative emotions and can have instantaneous impacts on mood.

#### Impact on Quality of Life

The participants described many ways in which they experienced socio-environmental pressures pertaining to their eating and body image that impacted their quality of life, defined as the ability to participate in and enjoy life events. They described having a negative body image and making or experiencing judgments about their food choices. For example, this participant decided to celebrate a “happy moment” with food, and because he judged the amount and type of food he was eating, he hid the experience from his daughter:

“I ate in the car to avoid my daughter seeing me eat this food that I tell her to stay away from. (…) I would LOVE to have been able to celebrate that moment WITH my daughter, with perhaps a better meal.” (Pt 22)

The participants also described feelings of exclusion due to their weight, such as exclusion from daily activities and from experiences that they perceived people have when their weight is in the normal weight range. This participant noted several ways in which she felt being overweight negatively impacted her life:

“It (overweight) does undermine your ability to attract men to date. It undermines your ability to get a better job. (…) You may even not be able to get a job in leadership in your community because people want to have a representation of their company in a positive way, and society views being skinny as a positive.” (Pt 12)

Another participant described struggling to engage in activities because of physical health limitations as a result of their weight:

“I want to be around long enough to see (my children's) children and live a quality and productive life. I want to be able to participate in sports and activities and not feel bad because I am running out of breath.” (Pt 3)

This participant tried to avoid activities due to his weight:

“I find myself trying to avoid family pictures or doing too much in public because I'm uncomfortable with the way I look.” (Pt 6)

The participants described difficulties with how clothes fit as well as the financial burden of binge eating. For this participant, these two factors were related:

“Binge eating impacts my budget as I'm constantly purchasing the wrong foods and more clothes to fit my growing body.” (Pt 3)

Taken together, the ways in which binge eating and obesity impact people's daily lives provide insights into the types of reasons people are motivated to change their behaviors. They also inform specific domains in which individuals may experience challenges to initiate or sustain behavior change, such as concerns with body image, avoiding people or activities, and finances. Understanding these impacts is beneficial for informing areas to leverage or build added support in interventions.

### Personas

**Figure 1:**
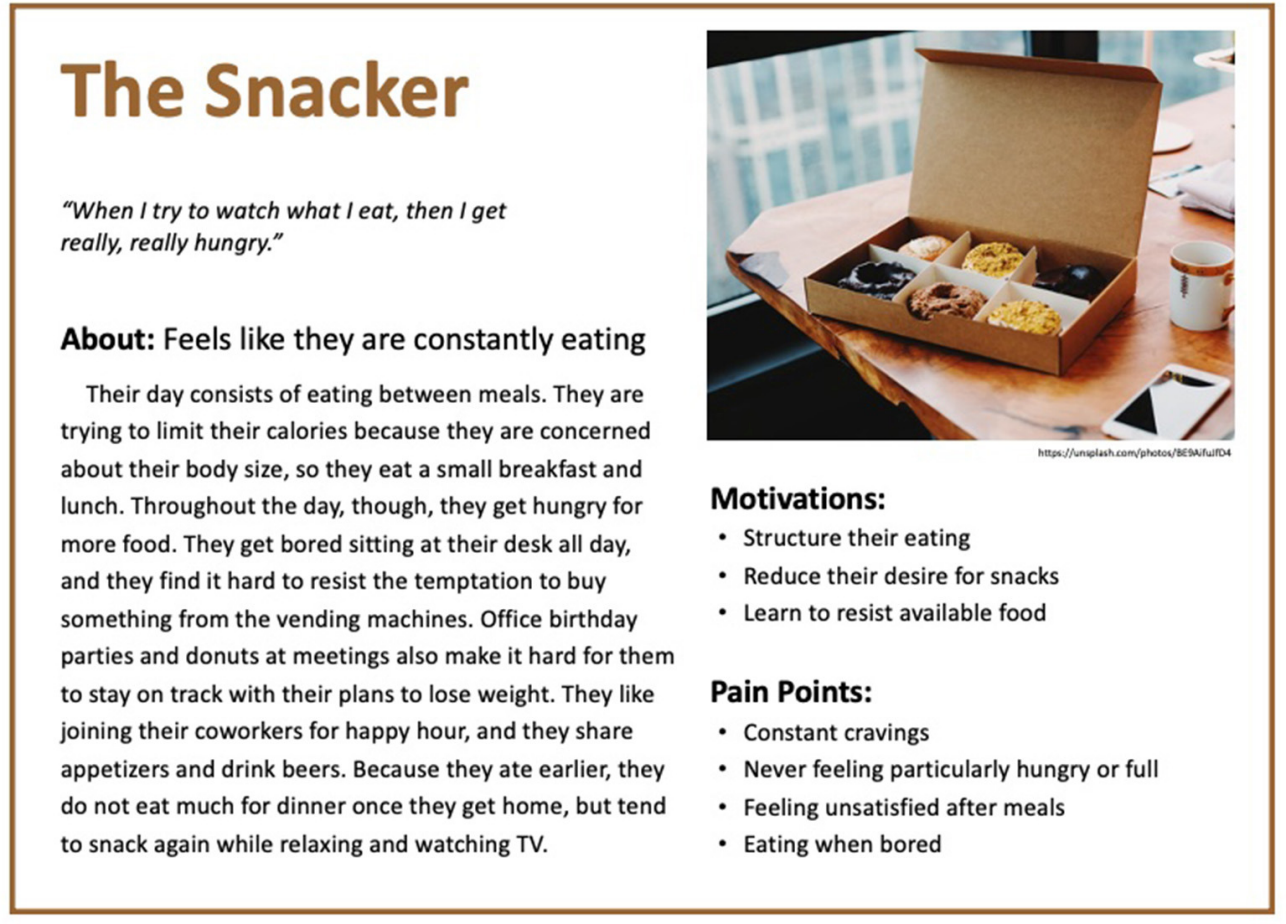
Persona 1: The Snacker.

**Figure 2:**
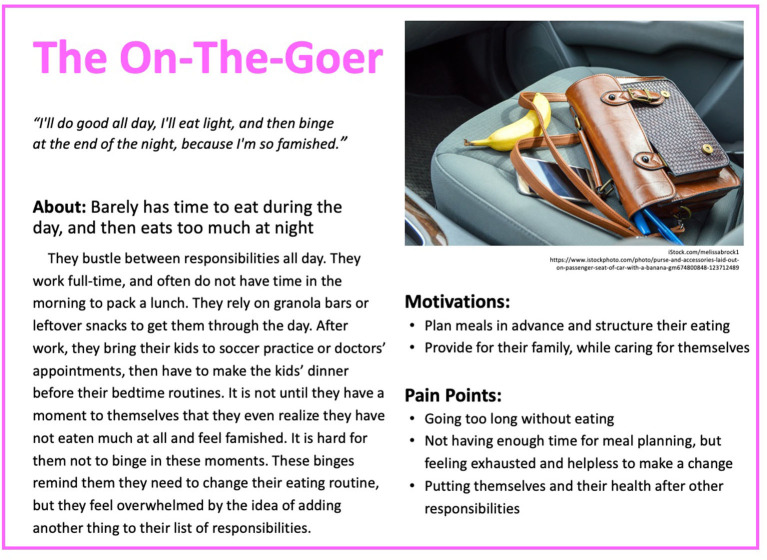
Persona 2: The On-The-Goer.

**Figure 3:**
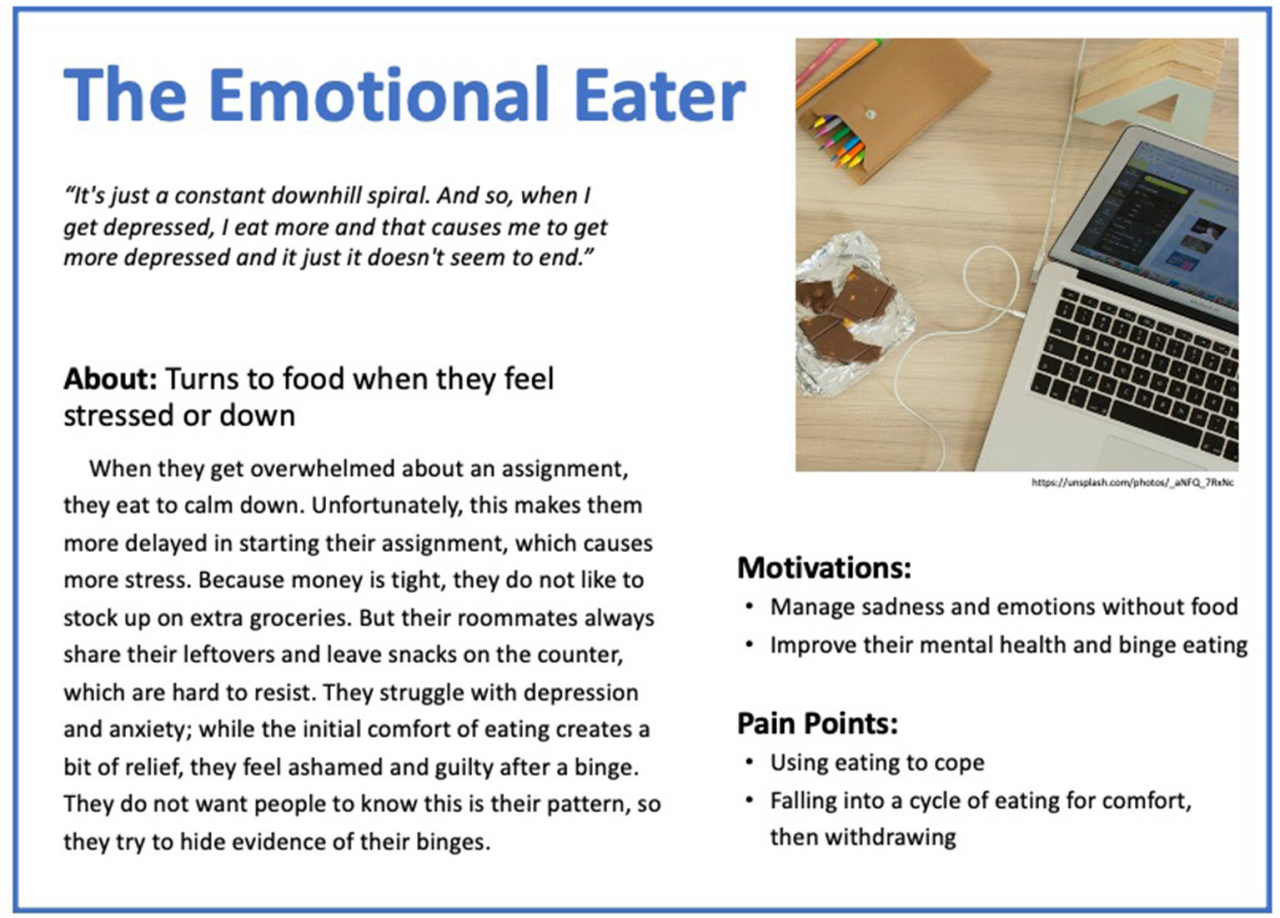
Persona 3: The Emotional Eater.

**Figure 4:**
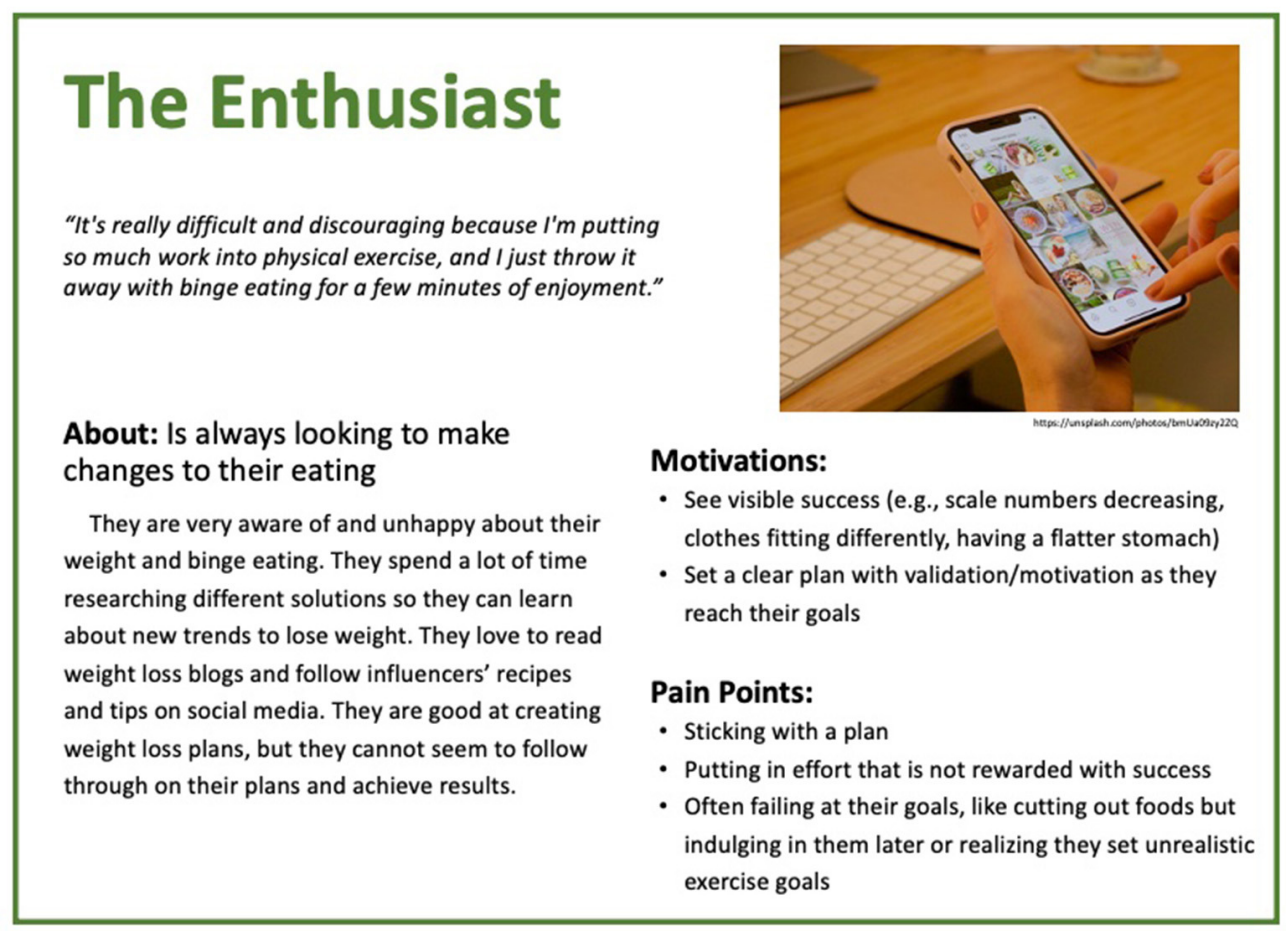
Persona 4: The Enthusiast.

**Figure 5:**
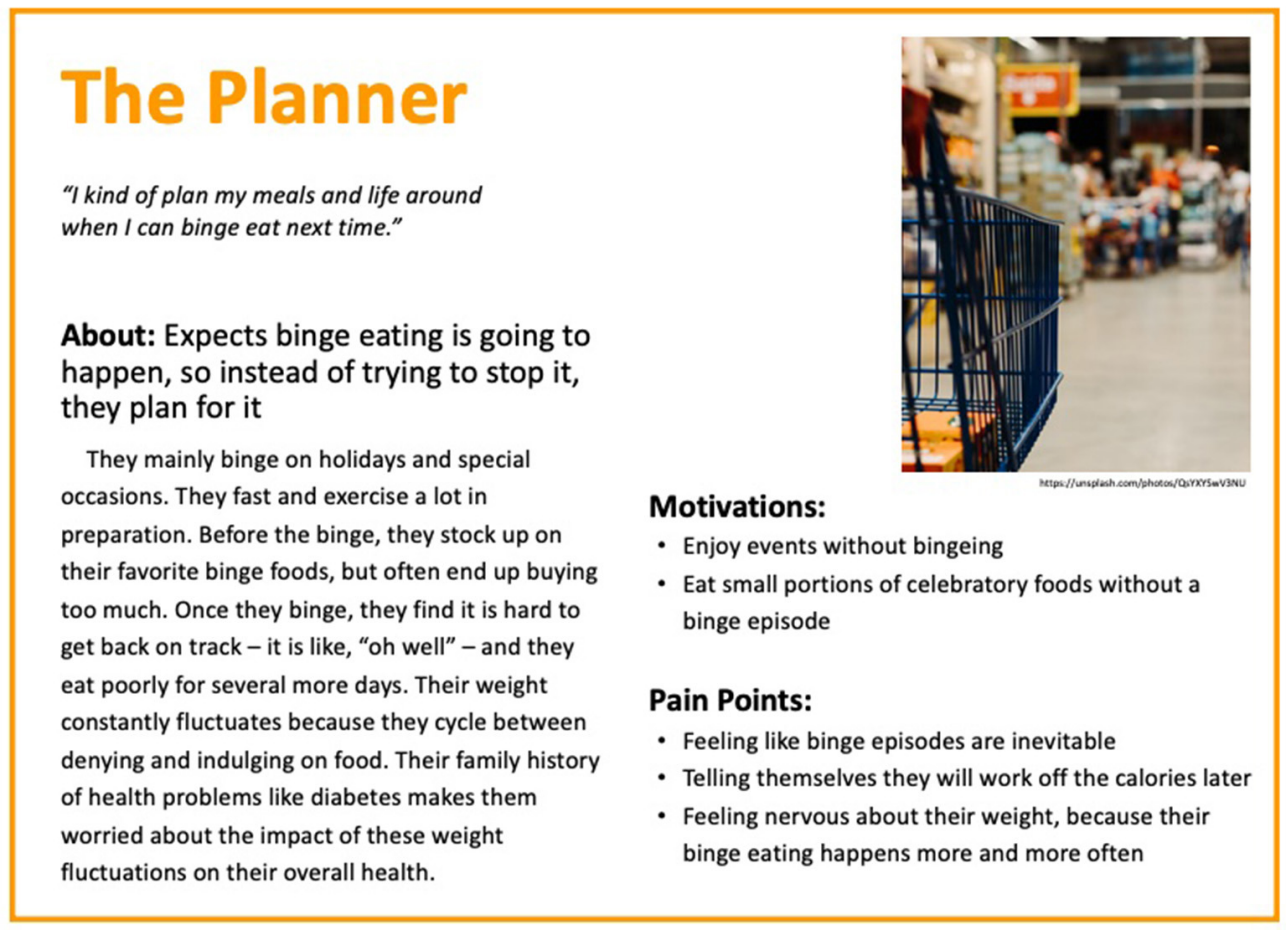
Persona 5: The Planner.

Based on these results, five personas were created to reflect the diversity of experiences of people with binge eating and obesity. The personas are depicted in Figures 1–5.

#### The Snacker

“*When I try to watch what I eat, then I get really, really hungry*.” The Snacker feels like they are constantly eating. Their day consists of eating between meals. They are trying to limit their calories because they are concerned about their body size, so they eat a small breakfast and lunch. Throughout the day, though, they get hungry for more food. They get bored sitting at their desk all day, and they find it hard to resist the temptation to buy something from the vending machines. Office birthday parties and donuts at meetings also make it hard for them to stay on track with their plans to lose weight. They like joining their coworkers for happy hour, and they share appetizers and drink beers. Because they ate earlier, they do not eat much for dinner once they get home but tend to snack again while relaxing and watching TV. They are motivated to change their behavior by creating structure to their eating, reducing their desire for snacks, and learning to resist available food. However, they struggle with having constant cravings, never feeling particularly hungry or full, feeling unsatisfied after meals, and eating when they are bored. They are aware that these factors impact their progress.

#### The On-The-Goer

“*I'll do good all day, I'll eat light, and then binge at the end of the night, because I'm so famished*.” The On-The-Goer barely has time to eat throughout the day and then eats too much at night. They bustle between responsibilities all day. They work full time and often do not have time in the morning to pack a lunch. They rely on granola bars or leftover snacks to get them through the day. After work, they bring their kids to soccer practice or doctors' appointments and then have to make the kids' dinner before their bedtime routines. It is not until they have a moment to themselves that they eventually realize that they have not eaten much at all and feel famished. It is hard for them not to binge in these moments. These binges remind them that they need to change their eating routine, but they feel overwhelmed by the idea of adding another thing to their list of responsibilities. They are motivated to change their behavior in terms of planning meals in advance, structuring their eating, and learning to provide for their family while caring for themselves. However, they recognize that progress will be challenged by their pattern of going too long without eating, not having enough time for meal planning, feeling exhausted and helpless to make a change, and putting themselves and their health after other responsibilities.

#### The Emotional Eater

“*It's just a constant downhill spiral. And so, when I get depressed, I eat more, and that causes me to get more depressed, and it just doesn't seem to end*.” The Emotional Eater turns to food when they feel stressed or down. When they get overwhelmed about an assignment, they eat to calm down. Unfortunately, this makes them more delayed in starting their assignment, which causes more stress. Because money is tight, they do not like to stock up on extra groceries, but their roommates always share their leftovers and leave snacks which are hard to resist on the counter. They struggle with depression and anxiety; while the initial comfort of eating creates a bit of relief, they feel ashamed and guilty after a binge. They do not want people to know that this is their pattern, so they try to hide the evidence of their binges. They want to change their behavior to no longer manage sadness and emotions with food and to improve their mental health and binge eating, yet currently, food is their coping tool, and they fall into a cycle of eating for comfort and then withdrawing.

#### The Enthuisiast

“*It's really difficult and discouraging because I'm putting so much work into physical exercise, and I just throw it away with binge eating for a few minutes of enjoyment*.” The Enthuisiast is always looking to make changes to their eating. They are very aware of and unhappy about their weight and binge eating. They spend a lot of time researching on different solutions so they can learn about new trends to lose weight. They love to read weight loss blogs and follow influencers' recipes and tips on social media. They are good at creating weight loss plans, but they cannot seem to follow through on their plans and achieve results. The Enthuisiast is very motivated to change their behavior. They want to see “visible success” (e.g., scale numbers decreasing, clothes fitting differently, having a flatter stomach) and to set a clear plan with validation/motivation as they reach their goals. Unfortunately, they find that, although they can organize a plan, they cannot stick with it, and they feel frustrated that they put in effort that is not rewarded with success. They often fail at their goals, like cutting out foods, by indulging in them later or realizing that they set unrealistic exercise goals.

#### The Planner

“*I kind of plan my meals and life around when I can binge eat next time*.” The Planner expects that binge eating is going to happen, so instead of trying to stop it, they plan for it. They mainly binge on holidays and special occasions. They fast and exercise a lot in preparation. Before they binge, they stock up on their favorite binge foods but often end up buying too much. Once they binge, they find that it is hard to get back on track—it is like, “oh well” —and they eat poorly for several more days. Their weight constantly fluctuates because they cycle between denying and indulging on food. Their family history of health problems like diabetes makes them worried about the impact of these weight fluctuations on their overall health. Because of this, the Planner wants to change their behavior and is motivated by the idea of being able to enjoy events without bingeing and eating small portions of celebratory foods without a binge episode, yet they feel that these changes are an uphill battle because they feel like binge episodes are inevitable. They tell themselves that they will work off the calories later but feel nervous about their weight because their binge eating happens more and more often.

## Discussion

Understanding the day-to-day experiences of individuals with binge eating and obesity helps inform intervention designs that can be relevant to diverse experiences. By conducting a qualitative needs assessment in real-world contexts, we gained an in-depth understanding of internal and external precipitants as well as consequences associated with obesity and binge eating. In turn, these findings informed the creation of five personas, archetypes of hypothetical users, which, by clarifying the different motivations and challenges behind the experiences of this population, provide a meaningful, tangible foundation to inform intervention design.

The findings from our digital diary study showed that individuals have varying precipitants of problematic eating and varying ways in which these health problems impact their lives, consistent with existing literature on these health problems [e.g., (6, 17)]. Importantly, however, our qualitative data were captured in the contexts of individuals' everyday lives. Methods like ecological momentary assessment have been used to capture self-reported in-context experiences of people with binge eating (43) but, to our knowledge, have been limited to quantitative data. Collecting in-context qualitative data allowed for an in-depth, narrative understanding of the ways binge eating and obesity manifest throughout the day, including the ways that these conditions are experienced cognitively and emotionally and how they are managed. Thus, our method of using a digital diary study offers another approach that clinical scientists can use to engage with end-users around understanding individual variations in precipitants, outcomes, and motivations.

Because individual variation exists, the challenge in creating a digital intervention is making a tool that applies to a broad catchment of users while being relevant to each user's individual needs and contexts. To help address this challenge, we created five personas that represent hypothetical users of our intervention. Based on the findings of our digital diary study, the personas demonstrate different motivations and barriers to behavior change that individuals experience in managing binge eating and weight. The use of personas is helpful for informing how a technology can be designed to meet the diverse needs of individuals. They can reduce self-referential design decisions (e.g., assuming that the user would have the same experiences and preferences as the designer) and help the designer maintain a focus on the specifics of the user's experience instead of the limitations and benefits of a particular technology (21, 44). They can also be used to represent and communicate the user's profile and needs to various stakeholders, including to users themselves who may prefer to reference a persona's hypothetical experience using a tool than to personalize their feedback [e.g., (45)]. Similarly, we have developed personas that represent profiles of users with obesity and binge eating and that can thereby help us design an intervention that is ultimately responsive to the specific and varied experiences of users with this health profile.

By capturing and distilling several aspects of the user experience, these personas can help us prioritize the requirements for the intervention product. For example, our personas capture a number of internal and situational factors that influence binge eating, and that must be accounted for in the design process. Whereas the “On-The-Goer” illustrates important situational factors that can precipitate binge eating, like time of day (e.g., propensities toward late-night binge eating) and busyness (e.g., failure to eat throughout the day can exacerbate binge eating), the “Emotional Eater” captures the ways that internal factors precipitate binge eating, such as stress or feeling down. Referencing these personas can allow the designers to keep these user experiences in mind as they prioritize design features. For instance, an intervention is likely to be successful if it not only provides the user with on-demand access to tools based on their perceived need (e.g., as might help the “Emotional Eater” in times of distress) but also pushes reminders and support to users when they most need it (e.g., which could help the “On-The-Goer” take time to eat). We have incorporated such features into our intervention in the form of push notifications to praise or encourage progress toward goals based on users' inputs into the app, along with delivering human coaching; these features enable providing tailored support to individual users' needs.

Our personas also remind us that interventions may be successful to the extent that they address multiple distinct types of users. For instance, a “Snacker” might need help to disrupt recurrent snacking, such as by finding new ways to deal with boredom and establishing a regular pattern of eating instead. By contrast, an “On-The-Goer” might require help in planning meals or need reminders to keep healthy food on hand for snacks. An “Emotional Eater” might benefit from tools that support healthier strategies for coping with emotions that do not involve food. We have designed our intervention to offer an array of psychoeducational material and tools to support such problem areas, including encouraging boredom-reducing activities, meal planning, and addressing negative emotions. As users engage with the intervention, we can begin to leverage data to inform how to tailor these features to specific user profiles to streamline the user experience as has been pursued in other digital health interventions [e.g., (46)]. In fact, although the personas presented here are distinct hypothetical users (consistent with the persona design), in practice, actual users may overlap across personas. Intervention designs that flexibly deliver features to address multiple challenge areas users face, as well as changes in users' needs over time, would likely help to reflect this reality and thus have heightened relevance to users.

As we have shown, these design decisions are grounded in the findings from the early user-centered design activities and can be expressed and preliminarily evaluated by referencing the personas; however, they need to be validated through user testing. The subsequent phases of the user-centered design process, which we are employing in this program of research, are to design and iteratively refine prototypes of the intervention in collaboration with end-users and, after the intervention is developed, test it in practice. Taking these steps will be critical for learning whether our design decisions, such as those described above, lead to a positive user experience and sustained engagement in the intervention. The “final” phase of validating the intervention through user testing in practice also affords the opportunity to learn from users ways to optimize the intervention over time, which thus continues the user-centered design process.

The strength of this study was applying user-centered design methods to engage with a diverse group of users to delve deeply into their daily experiences and inform the design of our mobile intervention. Moreover, the use of the digital diary study *via* the dscout platform enabled capturing rich qualitative insights in context with relatively low burden on both participants and researchers. However, limitations should be noted. First, the participants were limited to dscout users. Although a core premise of the user-centered design methodology is that the voices of a few individuals are assumed to speak for the broader population (47), the participants in this study may not reflect all individuals with obesity and binge eating who are interested in losing weight and reducing binge eating. We also acknowledge that this is a small sample size in the clinical science field, but it is consistent with sample sizes in design research in the field of human–computer interaction (38, 39). Second, we used self-reported data to identify eligible participants as having obesity and recurrent binge eating (i.e., using self-reported height, weight, and episodes of binge eating); however, these measurements were not confirmed *via* objective assessment, which can vary from self-report (19). Finally, inter-rater reliability between coders was not assessed, although we note that this measure is not consistently calculated in papers of qualitative data from the field of human–computer interaction (48).

In conclusion, this study applied user-centered design activities to understand the day-to-day experiences of individuals with binge eating and obesity. These efforts are beneficial for informing intervention designs that can be relevant to diverse experiences and account for variations in these factors. Our process of taking a user-centered design approach to designing a mobile intervention contrasts the typical process that clinical scientists have used to design digital health interventions, that is, typical designs may be based on clinical expertise but do not sufficiently account for the experiences and preferences of the intervention consumer. Accordingly, this paper helps extend the clinical science literature by demonstrating design methods that can help clinical scientists design interventions that meet the needs of and intend to successfully engage its intended consumers. Indeed because personas have not been created to represent people with both obesity and binge eating, our personas and the methods that we employed to create them are useful for informing the design of our own mobile intervention as well as the design of other behavioral interventions. As we have described, the insights from our digital diary study needs assessment and the resulting personas have been used to design prototypes and to confirm that the prototype iterations and the resulting mobile intervention being designed meet the needs, goals, and preferences of end-users.

## Data Availability Statement

The data that support the findings of this study are available from the corresponding author upon reasonable request. Requests to access the datasets should be directed to Andrea Graham, andrea.graham@northwestern.edu.

## Ethics Statement

The studies involving human participants were reviewed and approved by Northwestern University Institutional Review Board. The patients/participants provided their written informed consent to participate in this study.

## Author Contributions

AG designed the study. AG and SN collected and analyzed the data. AG, AC, and JL drafted the manuscript. EF, EG, RK, and JN critically reviewed the manuscript for important intellectual content. All authors read and approved the final manuscript.

## Conflict of Interest

The authors declare that the research was conducted in the absence of any commercial or financial relationships that could be construed as a potential conflict of interest.
